# Effective CO_2_ Thermocatalytic Hydrogenation with High Coke Resistance on Ni-CZ/Attapulgite Composite

**DOI:** 10.3390/molecules29194550

**Published:** 2024-09-25

**Authors:** Shumei Chen, Jiacheng Fu, Yonghui Peng, Lixing Liang, Jing Ouyang

**Affiliations:** 1 Hunan Key Lab of Mineral Materials and Application, Department of Inorganic Materials, School of Minerals Processing and Bioengineering, Central South University, Changsha 410083, China235611072@csu.edu.cn (J.F.);; 2 Engineering Research Center of Ministry of Education for Carbon Emission Reduction in Metal Resource Exploitation and Utilization, Central South University, Changsha 410083, China

**Keywords:** attapulgite based material, CO_2_ methanation, Ni catalyst, CZ solid solution

## Abstract

Converting CO_2_ into methane is considered a promising and economically viable technology for global transportation and utilization of this greenhouse gas. This study involves the preparation of a Ni-CZ (CeO_2_-ZrO_2_)/ATP (attapulgite) catalyst through the co-precipitation and impregnation methods. XRD, SEM, TEM, N_2_ absorption-desorption isotherms, XPS, H_2_-TPR, CO_2_-TPD, TG/DSC, and Raman were adapted to characterize the obtained samples. Real-time GC was used to measure the catalytic performances and to intensively study the impact of Ni loading content and ATP to CZ ratio on the catalytic performance of the products. DRIFTs was used to monitor the interstitial radicals in the catalytic reactions and to deduce the catalytic mechanisms. The results indicate that the composite catalytic matrix composed of CZ assembled on ATP demonstrated higher CO_2_ methanation stability and better carbon deposition resistance ability than the single CZ or ATP as the carrier, which should be attributed to the improved specific surface area and pore volume of the ATP assembled matrix and the enhanced dispersibility of the CZ and Ni species. The adoption of CZ solid solutions improves the oxygen storage capability of the catalyst, thereby providing continued mobile O^2−^ in the matrix and accelerating the molecular exchange rate in the catalytic reactions. The ideal loading quantity of nickel contents on the CZA matrix is 15%, as the CO_2_ conversion decreases at elevated temperatures when the Ni loading content reaches 20%. Among the tested samples, the 15Ni-0.8CZA sample showed the best catalytic performance of 75% CO_2_ conversion and 100% CH_4_ selectivity at 400 °C. After 50 h of stability tests, the CO_2_ conversion rate still remained 70.84%, and the CH_4_ selectivity obtained 97.46%. No obvious coke was detected according to the Raman spectra of the used catalyst. The in situ DRIFTS experiment showed that formate is the main intermediate of the CO_2_ hydrogenation reaction on the 15Ni-0.8CZA catalyst.

## 1. Introduction

Gas emissions from cars and factories have caused serious environmental problems. To address global climate change, it is necessary to reduce greenhouse gas emissions [1] through some specific technologies, such as converting them into value-added products. Methane is a very valuable and major energy source. In the past few decades, many studies have been conducted on the methanation process of carbon dioxide, which involves the use of various catalytic systems to produce methane [2,3]. Among them, methanation reaction is a method suitable for large-scale CO_2_ fixation. This method can be used to convert carbon dioxide into synthetic natural gas (methane), recover CO_2_ for fuel or chemicals, and help reduce carbon dioxide emissions [4].

Generally, an ideal CO_2_ conversion catalyst should contain the following: (1) certain kinds of active metal species (often including Ni, Co, Fe, Ru, Pd, etc., and their alloys or solid solutions [5,6,7,8]) for the absorption and activation of H_2_ and CO_2_; (2) an assistant reagent (usually composed of some valence changeable cations, such as CeO_2_, TiO_2_, MnO_2_, or V_2_O_5_ [9,10,11,12]) to provide sufficient mobile electrons (originating from the decomposed H_2_ molecules to yield active H•) and mobile oxygen ions (to form oxygen vacancies to facilitate CO_2_ capture through M-O-C bonds [13,14]); and (3) a stable and porous matrix to support the active metals and to help disperse the metal nanoparticles or single atoms [15,16]. Decades of in-depth research have shown that excellent methanation performance has been achieved with different noble metal catalysts [17]. However, from the industrial perspective, their applications are unprofitable and unsustainable, and, more importantly, precious metals are likely to be easily sintered at high temperatures. Nickel-based catalysts have been widely studied as substitutes for rare and precious metal catalysts because nickel metal reserves are relatively abundant and because nickel has a competitive price advantage compared to precious metals [18,19,20].

Cerium oxides have been subjected to widespread attention because of their excellent redox properties [21]. Research has shown that cerium dioxide can be used in the removal of high-intensity organic compounds from combustion pollutants and wastewater. However, the thermal stability of pure CeO_2_ is poor [22]; some metal oxides should be introduced into the lattice to improve the stability of cerium oxide at high temperatures. For example, adding ZrO_2_ can improve the high-temperature stability and catalytic activity of CeO_2_ [23]. The formed CeO_2_-ZrO_2_ (CZ) solid solution has high thermal stability and fairly high oxygen storage capacity (OSC), which is considered to be one of the most promising industrial catalytic carrier materials [24,25]. Numerous studies have focused on the preparation and application of CZ solid solution to improve their OSC or specific surface area (SSA) so as to improve their catalytic performances [22,26,27].

In the aspect of selecting a matrix of the catalyst, attapulgite (ATP) has been considered a suitable candidate due to its unique high absorption capacity, fibrous morphology, high activity, environmental friendliness, and low price [28,29]. It has been widely used as adsorbent, filler, coating, binder, flame retardant, and catalyst matrix [30,31]. Designing and evaluating an ATP-assembled composite catalyst for CO_2_ conversion should offer new opportunities to understand the effect of the mineral matrix on catalytic performance and to analyze the mechanism of the catalytic reactions.

In this work, we prepared a series of CO_2_ hydrogenation catalysts with different Ni loading amounts and different ratios of ATP to CZ using attapulgite as the catalyst carrier, CZ solid solution as the assistant agent, and Ni nanoparticles as the active metal. Detailed characterization was conducted on the phase structure and morphology characteristics of the carrier materials and related catalysts. The CO_2_ methanation performance of catalysts with different Ni content and ATP-CZ ratios was compared. Based on the relevant characterization results, the mechanism of the CO_2_ methanation reaction was analyzed, and the results showed that formate was the main intermediate in the methanation process on 15Ni-0.8CZA.

## 2. Results and Discussion

### 2.1. XRD Analysis

**Figure 1:**
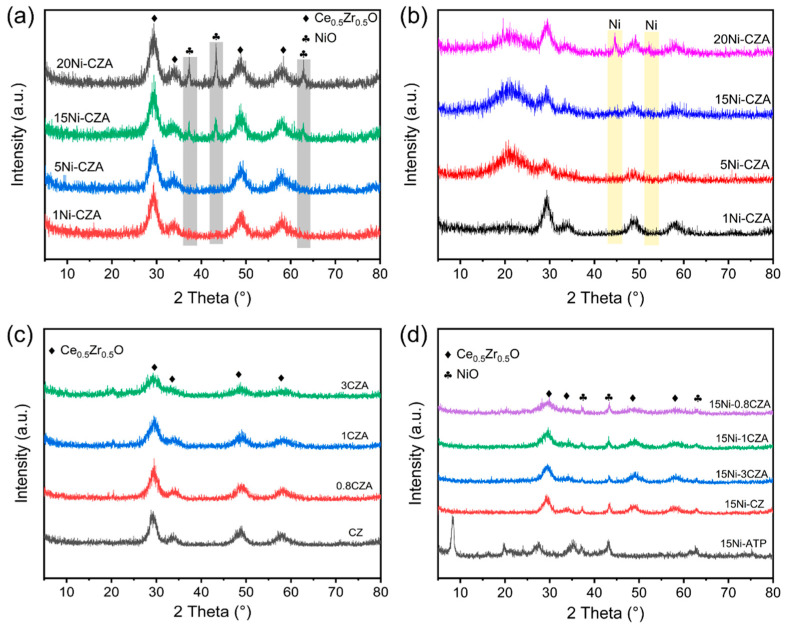
XRD patterns of catalysts with different Ni loading (**a**) before and (**b**) after reduction; XRD patterns of different ATP-CZ ratios catalysts, (**c**) series catalyst carriers, and (**d**) catalyst products.

Figure 1a has exhibited the XRD patterns of composite catalysts with different Ni loading before reduction; the peaks of CeO_2_-ZrO_2_ solid solutions can be observed in all catalyst samples, without attapulgite peaks. Peaks at 2*θ* = 37.4°, 43.4°, and 63° belonging to the NiO crystals can be observed. The intensity of the NiO peaks is weak with 1% and 5% Ni loading; the peaks of NiO can be clearly observed when the Ni loading increases to 15%, and the intensity is strongest at 20% Ni loading. After the 20Ni-CZA samples were reduced by H_2_ at 500 °C, diffraction peaks belonging to Ni can be observed at 2*θ* = 44.5°and 51.8° (Figure 1b) [32] with no NiO peaks, indicating that most of the NiO in the catalyst was reduced to Ni metal [33]. Figure 1c,d exhibits the XRD patterns of a series of catalyst carriers and products. Before loading (Figure 1c), the peak of typical Ce-Zr can still be observed in the 3CZA, 1CZA, and 0.8CZA samples, which are similar to their corresponding pure CZ solid solutions structure, and the peaks of attapulgite disappear because the attapulgite becomes amorphous phase due to the de-hydroxylation during the calcination process [34]. The high aspect ratio of the attapulgite matrix acts as a diffusion barrier during the calcination process, as well as hindering the growth of grain boundaries. Consequently, the crystallization performance of CZ oxides loaded on the concave clay surface is compromised [35]. This results in ambiguous diffractions in the XRD patterns of the 15Ni-ATP, 15Ni-CZ, 15Ni-3CZA, 15Ni-1CZA, and 15Ni-0.8CZA samples (Figure 1d). The peaks at 2*θ* = 37.4°, 43.4°, and 63° belonging to NiO crystals can be observed.

### 2.2. Pore Structure Analysis

**Figure 2:**
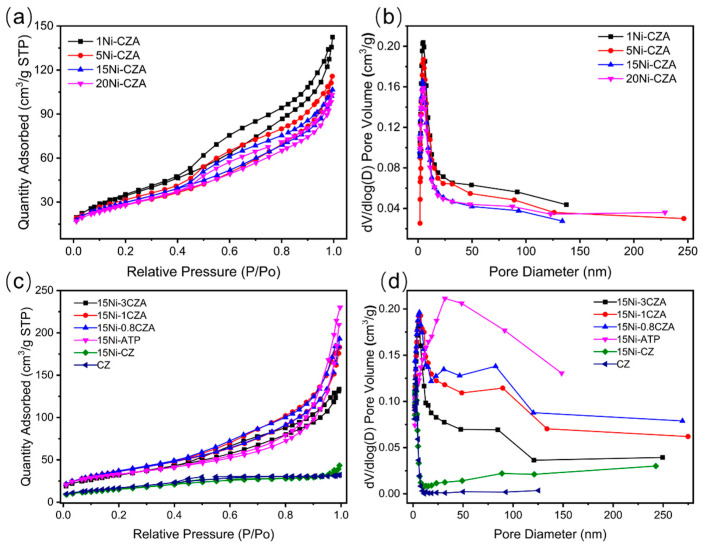
BET test results: (**a**,**b**) different Ni contents; (**c**,**d**) different ATP-CZ ratios.

Table 1 and Figure 2 show the BET tests of a series of products. According to Table 1, the order of specific surface areas of different Ni content catalysts is 1Ni-CZA > 15Ni-CZA > 20Ni-CZA > 5Ni-CZA. Samples 15Ni-CZA have larger specific surface areas. The variation order of pore volume is 1Ni-CZA > 5Ni-CZA >15Ni-CZA > 20Ni-CZA, which decreases with the improvement of Ni additive amount, possibly due to the aggregation of carriers and the filling of pores by Ni species [36]. The variation order of the average pore size is 5Ni-CZA > 20Ni-CZA > 1Ni-CZA > 15Ni-CZA. Samples 15Ni-CZA have smaller pore diameters, demonstrating that the thermal stability of the catalyst 15Ni-CZA is superior to that of 5Ni-CZA, 20Ni-CZA, and 1Ni-CZA.

Figure 2c shows the N_2_ adsorption and desorption isotherms of the sample 15Ni-3CZA, 15Ni-1CZA, 15Ni-0.8CZA, and 15Ni-ATP exhibit obvious mesoporous material characteristics. Table 1 shows that the specific surface area of pure cerium zirconium solid solutions is 63.32 m^2^/g. After loading Ni metal, the surface area was reduced to 58.30 m^2^/g. The addition of the Ni element further reduced the specific surface area of the sample, which was attributed to the blocking of the pore structure of the CZ after immersion in Ni solvent. The surface area of the catalyst assembled with CZ solid solution and attapulgite is higher than that of the pure cerium zirconium solid solution supported with Ni, which indicates that the composite catalyst prepared from cerium zirconium solid solution and attapulgite achieves a higher specific surface area than that of a single material and the use of cerium zirconium solid solution and attapulgite assembly is beneficial to improving the textural properties of the carrier. The specific surface area of catalyst 15Ni-0.8CZA is the highest among all samples (133.07 m^2^/g). Figure 2d shows the corresponding pore distribution curve. It can be observed that the pore size distribution of 15Ni-0.8CZA is broader, with an average pore diameter of 7.79 nm.

### 2.3. SEM and TEM Analysis

**Figure 3:**
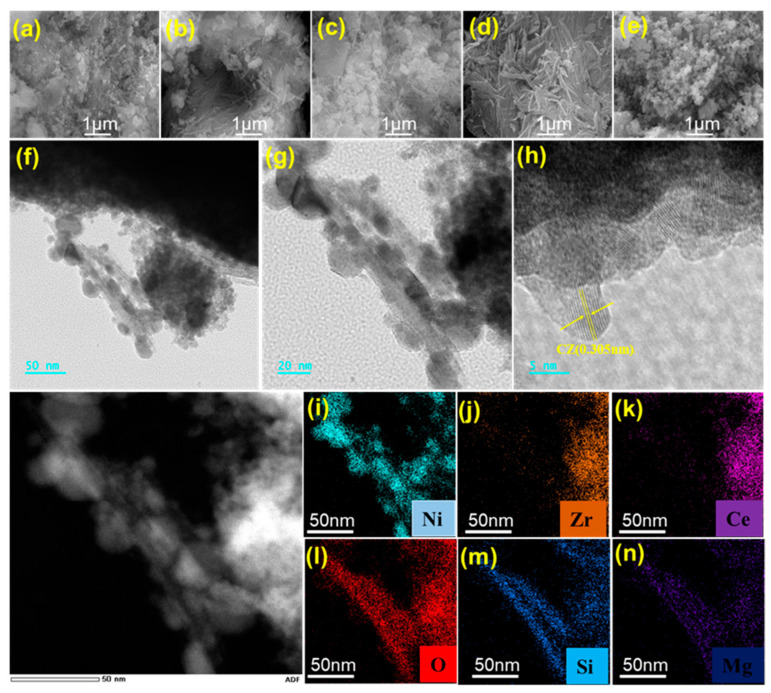
SEM diagram of catalysts (**a**) 15Ni-3CZA, (**b**) 15Ni-1CZA, (**c**) 15Ni-0.8CZA, (**d**) 15Ni-ATP, and (**e**) 15Ni-CZ; (**f**,**g**) TEM diagram, (**h**) HRTEM diagram, and (**i**–**n**) element mapping diagram of catalyst 15Ni-0.8CZA.

Based on the previous analysis, the 15% Ni loading has been determined as the suitable concerntration as active metals; therefore, this section focuses primarily on discussing the microscopic morphology of the 15% Ni series catalysts. The morphologies of catalysts 15Ni-3CZA, 15Ni-1CZA, 15Ni-0.8CZA, 15Ni-ATP and 15Ni-CZ are shown in Figure 3a–e. Catalyst 15Ni-CZ exhibits a blocky structure, and CZ solid solutions tend to reunite in high-temperature conditions. The typical irregular rod-shaped structure of attapulgite is evident in the 15Ni-ATP catalyst. Due to a parallel grouping behavior of this nanofibrous mineral, it tends to adhere and form bundles [37]. The morphology of attapulgite is barely discernible in 15Ni-3CZA. As the proportion of attapulgite increases, the rod-shaped morphology becomes visible in the catalysts 15Ni-1CZA and 15Ni-0.8CZA. Due to the barrier effect of attapulgite fiber, the aggregation of cerium zirconium solid solution particles during calcination can be effectively reduced, so very small particles can be observed [38]. The fibrous morphology of the attapulgite can be observed and is combined with CZ solid solution in Figure 3f,g. The lattice stripes with a distance of 0.305nm observed in Figure 3h correspond to the (111) lattice distance of CZ in HRTEM graphics [39]. Focusing on the 15Ni-0.8CZA catalyst with the best catalytic performance, Figure 3i–n shows that Ni is uniformly distributed over catalyst 15Ni-0.8CZA. In addition, Ce and Zr elements only exist in the cerium zirconium solid solution, while Mg and Si elements belong to the components in the attapulgite, and no other impurity components can be observed.

### 2.4. Catalytic Performances toward CO_2_ Methanation

**Figure 4:**
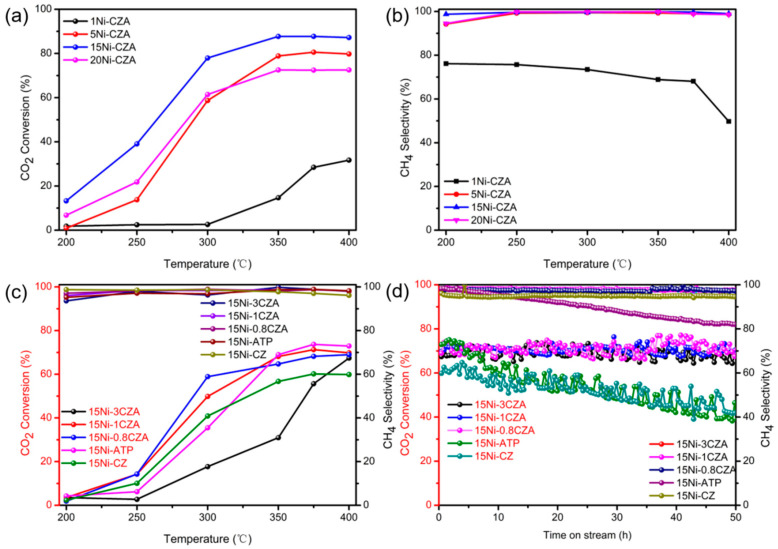
Catalyst activity test: (**a**,**b**) different Ni contents; (**c**) different ATP CZ ratios; (**d**) stability test results of different catalysts (50 h); Conditions: atmospheric pressure, CO_2_/H_2_/N_2_ = 1/4/5, GHSV = 48,000 mL/gh.

The catalytic activity and stability of different catalysts in CO_2_ methanation are shown in Figure 4. Prior to testing, the catalyst is reduced by H_2_ for 120 min. In Figure 4a, we present the results of a series of catalytic activity tests conducted at temperatures ranging from 200 to 400 °C. With the increase of Ni content from 0 to 15%, the conversion rate increases. When the Ni content reaches 20%, the conversion rate begins to decrease, and the CO_2_ conversion efficiency of catalyst 15Ni-CZA is the best. When the Ni loading amount is 20%, the CO_2_ conversion rate is not as good as 15Ni-CZA, indicating that the loading content of Ni should be controlled below 20%. This can be explained that, when the Ni content amount is too high, they will gather together to form large agglomerates, resulting in the poor dispersion of the carrier or, considering the catalytic mechanism, the intermediate formate apecies (will be discussed in the DRIFTs results) may accumulate on the dense nickel layer, resulting in the reduction of the effective surface area and porosity. Therefore, the optimal loading amount of the Ni-CZA catalyst selected is 15%. As shown in Figure 4b, except for the 1Ni-CZA catalyst, catalysts containing 5%, 15%, and 20% Ni species have shown much higher CH_4_ selectivity, approaching 100% under 400 °C. The Ni metal surfaces should interact strongly with composite carriers, and their dispersion on catalyst surfaces contributes to the strong interaction. As a result, the 15Ni-CZA catalyst exhibits the best CO_2_ methanation performance in CO_2_ methanation reactions.

Catalytic activity of samples containing different ATP-CZ ratios are shown in Figure 4c. As the reaction temperature rises, the CO_2_ conversion rates of the tested five catalysts gradually increase. Without the introduction of attapulgite, the CO_2_ conversion of 15Ni-CZ only reaches about 60% at 400 °C, while the catalyst 15Ni-ATP exhibits relatively prominent catalytic activity at 350 °C to 400 °C; the catalysts 15Ni-1CZA and 15Ni-0.8CZA also show good CO_2_ conversion rates, and the CO_2_ conversion rate can reach about 70% at 400 °C. The 15Ni-1CZA and 15Ni-0.8CZA catalysts also showed relatively excellent CH_4_ selectivity effects, which approached 100% at 400 °C. This may be originated from the evenly distributed Ni metal particles on the catalyst surface and interacts strongly with the carrier. To sum up, the 15Ni-0.8CZA catalysts have the best catalytic activity. 

Catalysts with different ATP-CZ ratios were further studied. There has a significant decrease in CO_2_ conversion on the 15Ni-ATP and 15Ni-CZ samples, the CO_2_ conversion rates dropped from 60% and 70% to 39.0% and 41.6%, respectively. The 15Ni-ATP also has a significant decrease in the CH_4_ selectivity. The CO_2_ conversion rates of 15Ni-3CZA, 15Ni-1CZA, and 15Ni-0.8CZA are 68.4%, 69.0%, and 70.8%, respectively, after 50 h of uninterrupted continuous tests. The CH_4_ selectivity were still remained at 97.3%, 97.4%, and 97.5% after 50 h tests, respectively, which indicates that the cerium zirconium solid solution assembled on attapulgite substrate has very good CO_2_ methanation stability, compared to the two single ATP or CZ alone as the carriers. In addition, the CO_2_ methanation stability of the composite catalyst is improved with the increase in ATP ratio. It shows that the combination of ATP and CZ as catalyst carriers can improve the performance and stability of the catalyst. Therefore, the catalyst 15Ni-0.8CZA has the most excellent catalytic activity.

As summarized in Table 2, compared with other catalysts, the series of 15Ni-CZA catalysts reported in this paper have obvious advantages in CO_2_ methanation performance and long-term stability, reflecting its promissing potential for industrial applications.

### 2.5. H_2_-TPR and CO_2_-TPD Analysis

**Figure 5:**
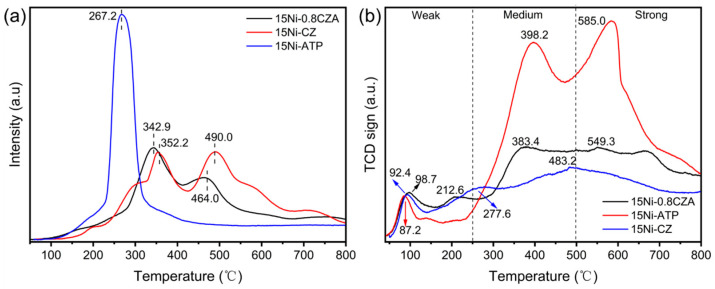
(**a**) H_2_-TPR and (**b**) CO_2_-TPD curves of the 15%Ni loadedon different matrix catalysts.

The H_2_-TPR curves of three catalysts are shown in Figure 5a. Table 3 shows quantitative data on hydrogen consumption. A small but strong peak centered at 352.2 °C and 342.9 °C is observed in 15Ni-CZ and 15Ni-0.8CZA, respectively, which can be attributed to the conversion of Ni^2+^ to Ni^0^ species [40]. The observed reduction peaks of 15Ni-CZ and 15Ni-0.8CZA in higher temperature regions (490.0 °C and 464.0 °C, respectively) can be attributed to NiO species having strong interaction with the carrier or assigned to the reduction of surface Ce^4+^ [44]. For the 15Ni-ATP catalyst, there is only a reduction peak at 267.2 °C, which may be due to a reduction in NiO species on the ATP carriers. Generally, there’s a weak interaction between NiO and the ATP carrier [45]. Compared to 15Ni-ATP, the 15Ni-CZ and 15Ni-0.8CZA samples exhibit higher reduction temperatures and two obvious reduction peaks, indicating that the addition of CZ solid solution can enhance the interfacial interaction between the active metal and the carriers, which effectively prevents the sintering of Ni particles under reaction conditions [46].

Figure 5b and Table 4 show the CO_2_-TPD test results of catalysts 15Ni-ATP, 15Ni-CZ, and 15Ni-0.8CZA. It is generally believed that CO_2_ desorption peaks below 250 °C, between 250 and 500 °C, and above 500 °C belong to weakly alkaline sites, moderately alkaline sites, and strongly alkaline sites, respectively [47]. It can be found that catalysts 15 Ni-ATP and 15Ni-0.8CZA present strong alkaline sites at about 585 °C and 549.3 °C, respectively, and the tightly adsorbed CO_2_ can react with CH_4_ at high temperature. In addition, CO_2_ is adsorbed on the catalyst by chemical adsorption, so there is a strong chemical bond type interaction between CO_2_ and the catalyst [48]. This may result in a small quantity of CH_4_ adsorbed on the active metal and promote the formation of carbon deposits at elevated temperature (up to 600 °C), leading to catalyst deactivation. The catalysts 15Ni-0.8CZA exhibit comparatively stronger alkaline sites compared to 15Ni-CZ, which can effectively promote the improvement of catalytic performance at relatively mild conditions (~400 °C) in this work [20].

### 2.6. XPS Analysis

**Figure 6:**
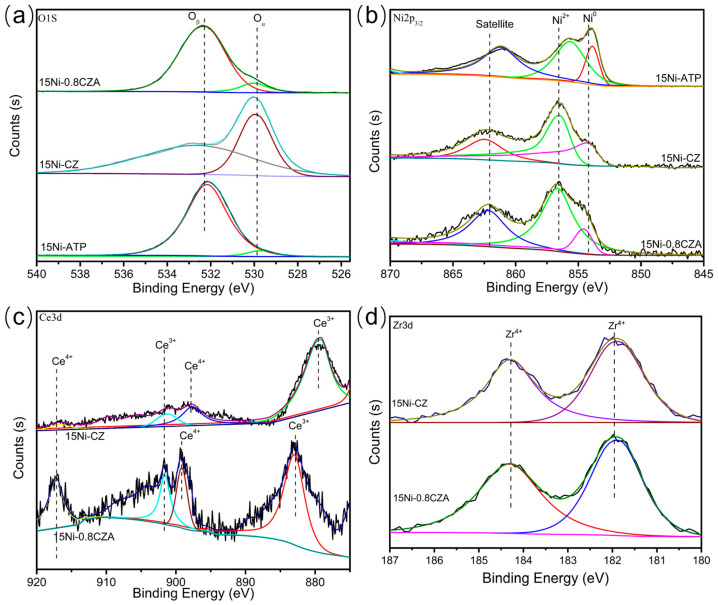
XPS spectra of 15Ni-ATP, 15Ni-CZ, and Ni-0.8CZA catalysts: (**a**) O 1s, (**b**) Ni 2p, (**c**) Ce 3d, and (**d**) Zr 3d.

Figure 6 shows the XPS test results of these catalysts for the purpose of delving into the elemental valence states and chemical bonding configurations between the optimal catalyst 15Ni-0.8CZA and the catalysts 15Ni-ATP and 15Ni-CZ. Regarding O 1s, it can be observed from Figure 6a that there are two peaks (529.3 eV and 532.4 eV) for O 1s in all samples. In particular, the shoulder peak at 532.4 eV is caused by surface O^2−^ anions binding to Ce^3+^ to maintain charge neutrality [39]. Figure 6b shows that the Ni 2p_3/2_ XPS profiles on all three catalysts exhibit similar Ni species categories, including Ni 2p_3/2_ (853.9 eV), which is accompanied by the satellite peaks and intensity of the main peaks of Ni 2p_3/2_ are similar. Interestingly, the Ni 2p_3/2_ peaks of these samples are accompanied by a shoulder peak centered at 856.5 eV. Unbound NiO has the Ni 2p_3/2_ peak at 854.0 eV [49], this means that NiO is present on the surfaces of catalysts 15Ni-ATP, 15Ni-CZ, and 15Ni-0.8CZA. In addition, the Ni 2p_3/2_ shoulder peak at 856.5 eV indicates that the binding energy of nickel is higher than that of unbound NiO. The catalyst 15Ni-0.8CZA has the strongest Ni 2p_3/2_ shoulder peak; this may be attributed to the strong interaction between NiO and 0.8CZA carriers, because the CZ cations on the surface may be replaced by Ni^2+^, which can help the catalyst to be more easily reduced. Figure 6c shows the XPS spectrum of Ce 3d. The XPS peak shapes of catalysts 15Ni-CZ and 15Ni-0.8CZA are similar. In general, the peaks at 880.8 eV, 885.3 eV, and 903.4 eV are pertain to Ce^3+^, while the peaks at 898.7 eV, 897.1 eV, and 916.8 eV belong to Ce^4+^ [50,51]. The co-existence of two types of Ce^4+^ and Ce^3+^ on the surface of these catalysts may endow them with excellent redox performance [44]. For the Zr 3d spectrum, from Figure 6d, it can be found that the Zr 3d peaks of catalysts 15Ni-CZ and 15Ni-0.8CZA are concentrated at about 182.0 eV and 184.3 eV. According to previous reports, this may be due to the presence of zirconia in the form of Zr^4+^ [44].

### 2.7. Characterization of the Used Catalysts

**Figure 7:**
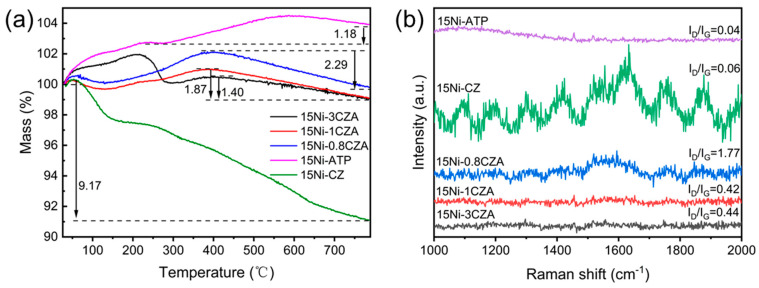
(**a**) TG curve and (**b**) Raman spectrum diagram of used catalysts.

The graphitization degree of the catalyst after long-term catalysis tests was characterized using thermogravimetric and Raman spectroscopy, as shown in Figure 7. As depicted in Figure 7a, the catalysts 15Ni-0.8CZA, 15Ni-1CZA, and 15Ni-3CZA exhibit a weight increase between 300 and 400 °C due to the oxidation of Ni. The weight loss after 400 °C is the combustion of surface carbon, indicating that the catalysts 15Ni-3CZA, 15Ni-1CZA, 15Ni-0.8CZA, 15Ni-CZ, and 15Ni-ATP all have varying degrees of carbon deposition. Among them, the weight loss phenomenon of 15Ni-CZ is the most obvious, indicating that CZ as a catalyst carrier alone has poor resistance to carbon deposition. The conclusion is consistent with the report by Xu et al. [44]. BET results also show that the assembly of CZ and ATP is beneficial for improving the texture properties of the carrier, thereby enhancing the long-term stability of the catalyst. Raman spectroscopy estimates the degree of graphitization through the I_D_/I_G_ ratio [47]. The Raman spectra of the five catalysts after testing are shown in Figure 7b. The I_D_/I_G_ ratios of catalysts 15Ni-3CZA, 15Ni-1CZA, 15Ni-0.8CZA, 15Ni-CZ, and 15Ni-ATP are 0.44, 0.42, 1.77, 0.06, and 0.04, respectively, the graphitization degree of catalyst 15Ni-0.8CZA is very low, indicating a very high coke resisstance ability of this sample.

### 2.8. Catalyst Reaction Mechanism Analysis

**Figure 8:**
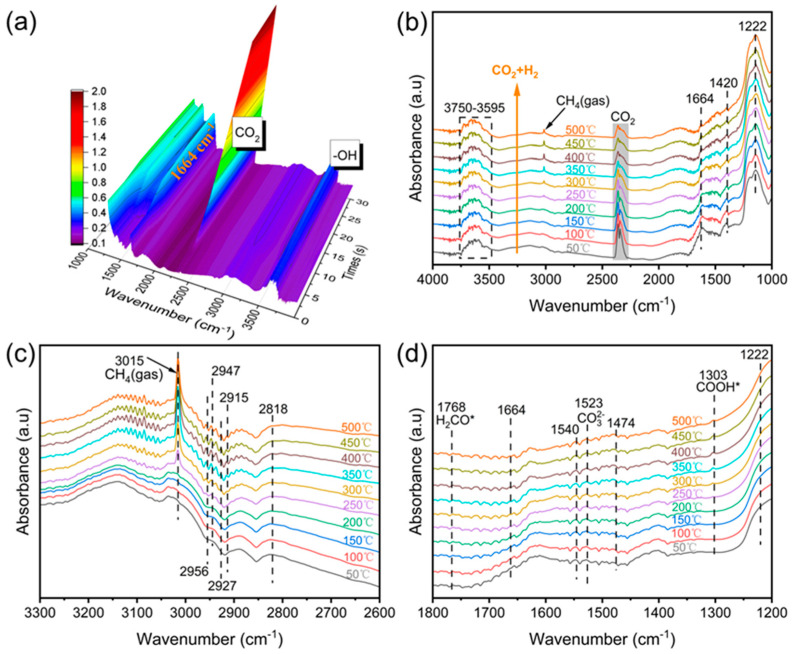
In situ DRIFTS spectra of catalyst 15Ni-0.8CZA: (**a**) IR spectra of different CO_2_ adsorption times; (**b**) real-time IR spectra of CO_2_ methanation at different temperature; (**c**) enlarged images of Figure 8b between 3300–2600 cm^−1^; (**d**) enlarged images of Figure 8b between 1800–1200 cm^−1^.

The DRIFTS test results of the 15Ni-0.8CZA catalyst are shown in Figure 8. Firstly, the adsorption of CO_2_ onto reduced samples was carefully messured. An adsorption saturation was detected after inflow of CO_2_ at 50 °C. The characteristic peaks of CO_2_ gas appear at 2300~2400 cm^−1^ after its introduction. When inspecting the change in signal strength of the CO_2_, the saturation was reached in about 10 s. Figure 8c,d are the enlarged spectra of Figure 8b within the selected wavenumber range. The stretching vibration peaks appear at 3015 cm^−1^, 2947 cm^−1^, and 2818 cm^−1^, which may be attributed to the C-H stretching in the methylene (=CH-) group, methyl (CH_3_-) group, and -CH_2_- group, respectively [52]. Thus, CHx- (x = 1, 2, 3) is an important intermediate for CO_2_ hydrogenation to CH_4_. As the reaction temperature increases, the absorbance of the =CH- group gradually increases at 3015 cm^−1^. This indicates that the generation rate of CH_4_ is fairly quick on the catalyst, which proves that 15Ni-0.8CZA has high catalytic activity for CO_2_ methanation [2]. The absorption peak of monodentate carbonate (CO_3_^2−^) is presented at 1523 cm^−1^, and the absorption peak of bicarbonate (*HCO_3_^−^) is presented at 1222 cm^−1^, indicating that monodentate formate and bicarbonate are potential reaction intermediates for activating CO_2_ [53,54].

**Figure 9:**
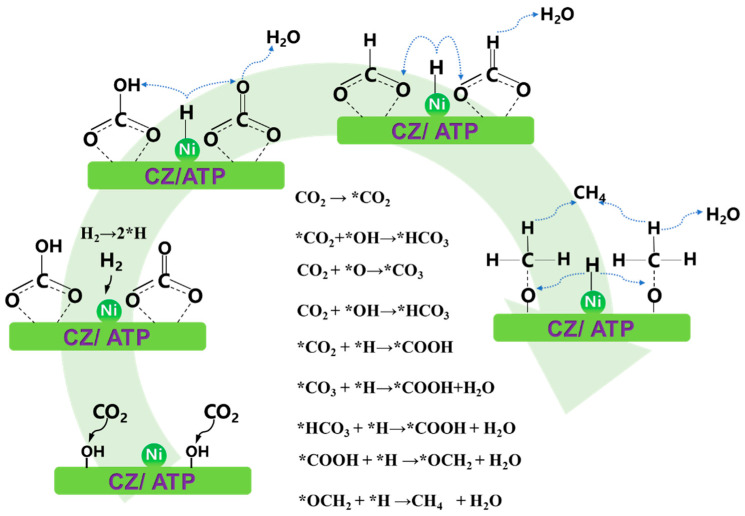
Possible reaction mechanism diagram of CO_2_ methanation on 15Ni-0.8CZA.

According to the above discussion, the reaction mechanism of the 15Ni-0.8CZA catalyst is in Figure 9. Hydrogen is firstly absorbed on the Ni site, then dissociates into *H, which overflows from the Ni site to the surface of the catalyst [55]. Carbon dioxide is first absorbed on the carrier surface, and some CO_2_ form the activated CO_2_ (marked as *CO_2_) [56]. The *CO_2_ reacts with the -OH on the surface of the catalyst. The *CO_2_ reacts with oxygen atoms to form bicarbonate [6]. Other inactive CO_2_ adsorbs on the catalyst surface and reacts with surface oxygen vacancies and surface hydroxyl groups to generate carbonates and bicarbonates, which react with *H to form formate. The *CO_2_ reacts directly with *H to form formate [57]. In the next step, the formate reacts with *H to generate *OCH_2_, which further reacts with hydrogen atoms to generate CH_4_ [58].

## 3. Experimental

### 3.1. Catalyst Preparation

**Figure 10:**
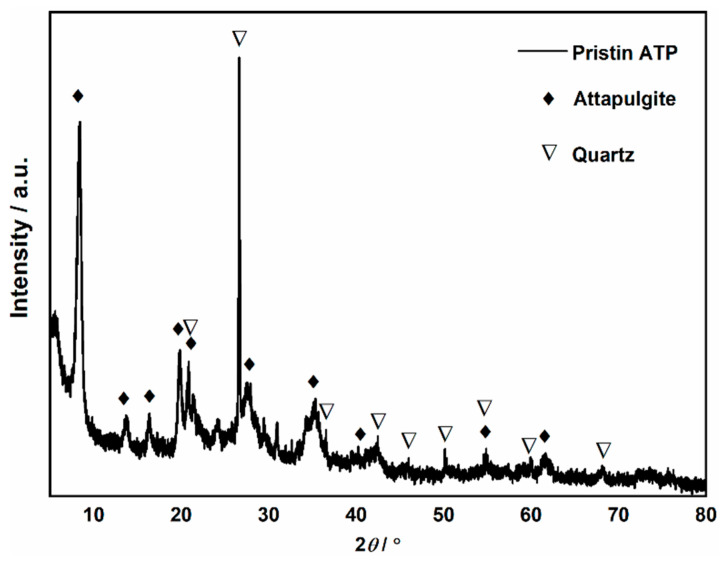
XRD patterns of the pristine ATP mineral.

The attapulgite was obtained from Xuyi, Jiangsu Province, and is recorded as ATP. The XRD pattern of the pristine ATP (Figure 10) exhibits characteristic peaks at 2*θ* = 8.38°, 13.83°, and 19.86°, which should belong to the stereotype attapulgite (JCPDS No. 29-0855) phase in the sample. Additionally, a sharp and singular diffraction peak corresponding to quartz crystals is observed at 2*θ* = 26.68°; some other diffractions that emerged on the pattern confirmed the co-existence of these two minerals. The SiO_2_ content is 62.47 wt.%, and the MgO content is 14.95 wt.%, according to the XRF results in Table 5.

**Figure 11:**
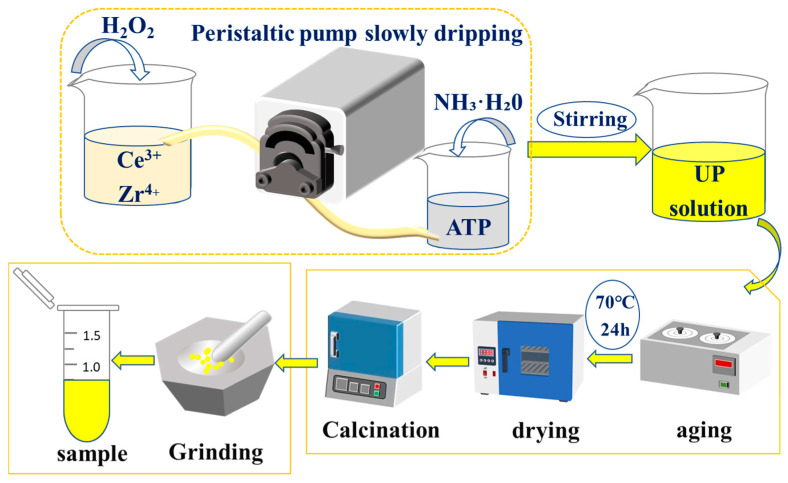
Preparation flow chart of the series of CZA catalysts.

The synthesis method of composite carrier materials is similar to our previous work and will not be repeated here. Figure 11 has shown the process; the specific preparation process is consistent with another paper by our team [38]. The ratio of cerium zirconium to attapulgite is 6:1. Then, corresponding nickel nitrate hexahydrate (Ni(NO_3_)_2_·6H_2_O) are added in weight percentages of 1%, 5%, 15%, and 20%, respectively. The mixtures are individually named as 1Ni-CZA, 5Ni-CZA, 15Ni-CZA, and 20Ni-CZA, according to Table 6.

In addition, composite catalysts with the designed matrices with different clay and cerium zirconium contents were prepared according to Table 6, where Ni was loaded with a settled weight percentage of 15% using the impregnation method. After drying and calcining, the obtained sample is grounded and collected for characterization and performance tests.

### 3.2. Catalytic Activity Test

Gas detection was performed using a Thermo Scientific Trace 1300 gas chromatograph (GC) with a catalyst dosage of 100 mg each time. Firstly, the catalyst was reduced in a H_2_ atmosphere (400 °C) for 120 min. Then, the temperature was decreased to room temperature, and a mixture of CO_2_ and H_2_/N_2_ (10% H_2_ and 90% N_2_) was introduced. After gradually increasing the temperature, the performance of the catalyst at 250–400 °C was measured according to the following calculations; the conversion rate of CO_2_ and CH_4_ selectivity was deduced according to the following equations [59]:

(1) $${\mathrm{CO}}_{2}\mathrm{Conversion}\left(\%\right)=\left(1-\frac{F_{{\mathrm{CO}}_{2},out}}{F_{{\mathrm{CO}}_{2},in}}\right)\times 100\%$$

(2) $${\mathrm{CH}}_{4}\mathrm{Conversion}\left(\%\right)=\left(\frac{F_{{\mathrm{CH}}_{4},out}}{F_{\mathrm{CO},\mathrm{out}}+F_{{\mathrm{CH}}_{4},out}}\right)\times 100\%$$

where $F_{{\mathrm{CO}}_{2},out}$ and $F_{{\mathrm{CO}}_{2},in}$ are the inlet and outlet dosages of the reactor, respectively; $F_{\mathrm{CO},\mathrm{out}}$ is the molar flow rate of the “CO” export product, and $F_{{\mathrm{CH}}_{4},out}$ is the molar flow rate of the “CH_4_” export product.

### 3.3. Catalyst Characterization Methods

The crystal phase of the samples was analyzed using a TD-3500 (the 2θ scanning range between 5 and 80°) diffractometer (Dandong Tongda Technology Co., Ltd., Dandong, China). The specific surface area of the sample was measured using a Micromeritics ASAP 2020 Plus HD88 (Mack Instruments, Norcross, GA, USA) physical adsorption analyzer at −196 °C, and pore size distribution data were obtained. The Titan G2 60-300 transmission electron microscopy (TEM, operated at 300 kV) (United States FEI Corporation, Peabody, MA, USA) and TESCANMIRA3LMU scanning electron microscopy (SEM, before testing, it is necessary to spray the sample with gold to improve its conductivity) (Czech company TESCAN, Brno, Czech Republic) were used to observe the morphology of the raw materials and obtained samples. The Auto Chem II 2920 chemical adsorbent (Mack Instruments, Norcross, GA, USA) was used for CO_2_ temperature programmed desorption (CO_2_-TPD) and H2 temperature programmed reduction (H_2_-TPR) tests. A NETZSCH STA-449C thermogravimetric (TG)differential scanning clorimetry analyzer (NETZSCH Scientific Instruments Trading (Shanghai) Co., Ltd., Shanghai, China) was used to measure the thermal stability of the precursor and possible carbon contents in the samples after long-term performance tests; the used catalyst (about 5–10 mg) is filled into an alumina crucible and heated from room temperature to 800 °C at a rate of 10 °C/min, and its mass reduction is calculated.

## 4. Conclusions

In conclusion, the catalysts with different Ni loading content (1Ni-CZA, 5Ni-CZA, 15Ni-CZA, and 20Ni-CZA) and different ratios of attapulgite to CZ (15Ni-3CZA, 15Ni-1CZA, 15Ni-0.8CZA, 15Ni-ATP, and 15Ni-CZ) were prepared by co-precipitation and impregnation methods for the methanation of CO_2_. The results indicate that the optimal loading amount of Ni is 15%, which may be because when the Ni loading amount is too high, it will cause the active component Ni to gather together, resulting in agglomeration on the support, thereby reducing the catalytic activity. The composite catalytic substrate material of attapulgite and CZ has better CO_2_ catalytic hydrogenation stability and superior carbon deposition resistance than the two materials as carriers alone. This is because the loading of attapulgite improves the specific surface area and pore structure of the catalyst, and the addition of a solid solution improves the redox characteristics of the catalyst, thereby accelerating the catalytic reaction. This suggests that the composite carrier of concave soil and CZ has a high potential as a carrier for Ni catalysts in methanation reactions. Among them, 15Ni-0.8CZA has the best catalytic effect. Mechanism analysis reveals that CO_2_ usually binds to oxygen vacancies and hydroxyl groups in the carrier, and Ni sites dissociate hydrogen into hydrogen atoms, with formate being the main reaction intermediate. This study provides theoretical guidance for the development of high-performance CO_2_ methanation catalysts from a carrier design and performance improvement perspective.

## Figures and Tables

**Table 1:** Specific Surface Area, Pore Volume, and Adsorption Average Pore Diameter of different samples.

Sample	*S_BET_* (m^2^/g)	*V_pore_* (cm^3^/g)	*D_pore_* (nm)
1Ni-CZA	127.02	0.21	5.40
5Ni-CZA	100.47	0.18	5.64
15Ni-CZA	108.32	0.16	5.18
20Ni-CZA	103.17	0.16	5.50
15Ni-3CZA	119.05	0.21	6.08
15Ni-1CZA	132.33	0.28	7.52
15Ni-0.8CZA	133.07	0.30	7.79
15Ni-ATP	116.74	0.33	11.63
15Ni-CZ	58.30	0.07	4.20
CZ	63.32	0.05	3.10

**Table 2:** Comparison of long-term stability of CO_2_ methanation with different catalysts.

Samples	Temperature (°C)	Time on Stream (h)	CO_2_ Conv. (%)	CH_4_ Sel. (%)	Ref.
45Ni/Ni005CZO	350	100	54	approximately100	[20]
Ni/CZ-AE	275	70	55	99.8	[40]
8Ni-TiO_2_-ATP	400	80	55	80	[41]
Ni/Zr/CNT-COI	350	50	≈40	95	[42]
Ni/Si	350	50	59.0	95.4	[43]
se-Ni/Sr/Si	350	50	65.5	97.7	[43]
se-Ni/Ba/Si	350	50	59.7	97.7	[43]
15Ni-3CZA	400	50	68.4	97.3	This work
15Ni-1CZA	400	50	69.0	97.4	This work
15Ni-0.8CZA	400	50	70.8	97.5	This work

**Table 3:** The H_2_-TPR quantitative results of the three 15% Ni series catalysts.

Samples	Peak1/°C	Quantity (mmol/g)	Peak2/°C	Quantity (mmol/g)
15Ni-ATP	267.2	3.35022	-	-
15Ni-CZ	352.2	2.19577	490.0	2.89357
15Ni-0.8CZA	342.9	1.93512	464.0	1.93321

**Table 4:** The CO_2_-TPD results of catalysts 15Ni-ATP, 15 Ni-CZ, and 15Ni-0.8CZA.

Samples	Peak1/°C	Quantity (mmol/g)	Peak2/°C	Quantity (mmol/g)
15Ni-ATP	87.2	0.09601	398.2	0.88576
15Ni-CZ	92.4	0.05382	277.6	0.15366
15Ni-0.8CZA	98.7	0.10319	212.6	0.10898
**Samples**	**Peak3/°C**	**Quantity (mmol/g)**	**Peak4/°C**	**Quantity (mmol/g)**
15Ni-ATP	585.0	1.30504	-	-
15Ni-CZ	483.2	0.52515	-	-
15Ni-0.8CZA	383.4	0.43050	549.3	0.68940

**Table 5:** Chemical composition of the pristine ATP sample (wt.%).

SiO_2_	MgO	Al_2_O_3_	Fe_2_O_3_	CaO	K_2_O	P_2_O_5_	TiO_2_	Na_2_O	MnO	Others
62.47	14.95	10.58	6.21	1.40	1.29	1.07	0.95	0.87	0.11	0.05

**Table 6:** Amount and proportion of metal, attapulgite, and cerium-zirconium solid solution required for preparation of target samples.

Samples	Ni (NO_3_)_2_•6H_2_O/g	CZA/g	CZ:ATP	Ni:CZ:ATP
15Ni-3CZA	0.25	0.5	3:1	0.15:3:1
15Ni-1CZA	0.25	0.5	1:1	0.15:1:1
15Ni-0.8CZA	0.25	0.5	0.8:1	0.15:0.8:1
15Ni-CZ	0.25	0.5	1:0	0.15:1:0
15Ni-ATP	0.25	0.5	0:1	0.15:0:1
1Ni-CZA	0.016	0.5	6:1	0.01:6:1
5Ni-CZA	0.083	0.5	6:1	0.05:6:1
15Ni-CZA	0.25	0.5	6:1	0.15:6:1
20Ni-CZA	0.33	0.5	6:1	0.20:6:1

## Data Availability

Data will be provided on demanding.
